# A Prospective Follow-Up of Adipocytokines in Cohort Patients With Gout: Association With Metabolic Syndrome But Not With Clinical Inflammatory Findings: Strobe-Compliant Article

**DOI:** 10.1097/MD.0000000000000935

**Published:** 2015-07-02

**Authors:** Sergio García-Méndez, Carolina Bustos Rivera-Bahena, José Luis Montiel-Hernández, Daniel Xibillé-Friedmann, Everardo Álvarez-Hernández, Ingris Peláez-Ballestas, Rubén Burgos-Vargas, Janitzia Vázquez-Mellado

**Affiliations:** From the Servicio de Reumatología, Hospital General de México, México City (SG-M, EA-H, IP-B, RB-V, JV-M); Dirección de Planeación, Enseñanza e Investigación. Hospital Regional de Alta Especialidad de Oaxaca, San Bartolo Coyotepec, Oaxaca (SG-M); Facultad de Farmacia, Universidad Autónoma del Estado de Morelos (CBR-B, JLM-H); Servicio de Reumatología, Hospital General de Cuernavaca “Dr. José G. Parres,” Cuernavaca, Morelos (DX-F); and Facultad de Medicina, Universidad Nacional Autónoma de México, México City, Mexico (IP-B, RB-V, JV-M).

## Abstract

The aim of this study was to determine the levels of leptin (Lep) and adiponectin (AdipoQ) in patients with gout and its relationship with joint inflammatory data and/or with metabolic syndrome (MetS) variables, during 1 year follow-up.

Forty-one patients (40 males) with gout diagnosis, attending for the first time to a rheumatology department, were included. Evaluations were performed baseline, at 6 and 12 months. Variables included the following: demographic, clinical and laboratory data related to gout and associated diseases. Lep and AdipoQ determinations by the ELISA method were performed in frozen serum from each visit. The pharmacological and no-pharmacological treatment for gout and associated diseases was individualized for each patient according to published guidelines. Statistical analysis included Mann–Whitney *U* test, Fisher test, *x*^2^, ANOVA, Cochran Q, Pearson and Spearman correlation tests, as well as linear regression.

In the baseline evaluation, 29.2% had MetS (hypertriglyceridemia 66%, hypertension 44% and obesity 37%); patients with MetS had higher C reactive protein (CRP) levels [34.1 ± 28.6 vs. 12.2 ± 11.2 mg/dL, *P* = 0.033]. Although not significant, also had higher Lep and lower AdipoQ levels (3.2 ± 3.0 vs. 1.9 ± 1.2 ng/mL, *P* = 0.142 and 40.5 ± 26.8 vs. 38.0 ± 24.9 ng/mL, *P* = 0.877, respectively). During follow-up, our patients had significant improvement in serum uric acid (sUA) levels and variables evaluating pain and joint swelling (*P* ≤ 0.05). Metabolic abnormalities tended to persist or even worsen during the monitoring period: significant increase in total cholesterol (*P* = 0.004), tendency to higher triglycerides (*P* = 0.883) and slight improvement in glycaemia (*P* = 0.052). Lep values increased significantly during follow-up (*P* = 0.001) while AdipoQ levels diminished slightly (*P* = 0.317). Neither Lep nor AdipoQ values showed important correlation (*r* > 0.5) with metabolic variables or joint swelling.

This study suggests that in patients with gout, concentrations of Lep and AdipoQ are more in line with the metabolic state than with clinical disease activity.

## INTRODUCTION

Adipocytokines are proteins secreted by white adipose tissue, among which leptin (Lep) and adiponectin (AdipoQ) have been associated with regulatory functions on energy metabolism and implicated as mediators of systemic inflammatory responses as well as promoting a “low degree” inflammatory status and mediating immune responses.^1–3^

These 2 adipocytokines are closely related to obesity and/or resistance to insulin and are important in the development of metabolic syndrome (MetS) and its components.^4–6^ In this context, a positive correlation between serum uric acid (sUA) and Lep levels has also been reported,^7–9^ as well as negative correlation with AdipoQ.^10,11^

The role that they play in different rheumatic diseases has recently been under study and, in spite of inconsistent results; a proinflammatory role has been described in osteoarthritis (OA) and rheumatoid arthritis (RA).^12–16^

In RA, results have been contradictory, as Lep levels may be higher than in healthy controls^12^ or even similar,^13^ related to the severity of RA^17–19^ and the serum/synovial fluid, Lep gradient is related to the time since onset of disease and disease activity.^20^ On the other hand, Lep has a negative correlation with radiological damage^21^ and in fasting conditions, may promote an antiinflammatory milieu.^22^ High concentrations of AdipoQ are promoters of the expression of inflammatory molecules;^14,23^ however, their presence has also been related to the inhibition of inflammatory pathways and immune response mediated by tumor necrosis factor α (TNFα) action.^24^

With regard to OA, it has been recently shown that joint tissue secretes greater amounts of Lep and this has been related to greater structural damage mediated by proinflammatory cytokines and matrix metalloproteinases.^12,15,25–27^ The role of AdipoQ in OA is controversial; on the one hand, its serum concentration is significantly higher than in healthy controls,^28^ related to severity^29^ and joint inflammation,^30^ while on the other hand there is evidence that links it with less clinical and radiological damage.^31^

There are few studies in patients with gout in which the role of these adipocytokines had been studied,^32–34^ documenting a significant increase of AdipoQ in patients who received benzbromarone;^32,34^ apparently, this increase in AdipoQ is produced by an agonist effect on peroxisome proliferator-activated receptors gamma (PPARγ) with anti-inflammatory consequences.^32^ In a case control study it was observed that the serum concentrations of Lep and AdipoQ were related to the body mass index and the amount of abdominal fat.^33^ It must be pointed out that none of these studies evaluated the relationship between these adipocytokines and the reduction in joint inflammatory activity of patients.

The objective of the present study was to determine the levels of Lep and AdipoQ in patients with gout under regular treatment and its relationship with the presence of MetS and joint inflammatory activity.

## METHODS

This was a prospective cohort study (GRESGO (GRupo de EStudio de GOta) that includes patients with gout diagnosis (according to ACR and CGD criteria),^35,36^ whom attended to the rheumatology department of our hospital for the first time. The sample size was no-probabilistic.

The project was approved by the local ethics and research committees and in the baseline visit all patients signed an informed consent form.

They were evaluated clinically by 1 of the participating rheumatologists (S.G.-M., E.A.-H., or J.V.-M.) and received verbal and written information about gout and associated diseases.

The prescription in the baseline and subsequent visits included the following: lifestyle modifications: diet, exercise, and weight reduction, and pharmacological treatment including allopurinol or other urate-lowering drug (ULD), nonsteroidal antiinflammatory drugs (NSAID), steroids, colchicines, and treatment for associated diseases according to published recommendations for gout treatment.^37,38^

The visits were performed as frequent as the clinical status of each patient required, but all them attended at least to the baseline, 6 and 12 months visits. The evaluation included tender, swollen, and limited to motion joint count; the number of acute flares in the last 6 months, clinimetric evaluation (HAQ questionnaire, VAS for pain and global health).

During the patient regular visit to the laboratory for biochemical determinations, 1 mL of serum was frozen at −70°C until adipocytokine determination. It was performed through indirect ELISA using a human anti-leptin antibody (Santa Cruz Biotech, Inc.), human recombinant leptin (PeproTech, Inc.), antiadiponectin antibodies (Santa Cruz Biotech, Inc.), and human recombinant adiponectin (R&D Systems). All of the determinations were done in triplicate in the 3 evaluations and were reported as means and standard deviations.

The presence of MetS and its associated entities was determined according to the criteria proposed by the Adult Treatment Panel III (ATP III):^39^ obesity (waist circumference ≥88 cm in women or ≥102 cm in men), dyslipidemia (high density lipoprotein-cholesterol (HDL-C) ≥40 mg/dL in men or ≥50 mg/dL in women, and triglycerides ≥150 mg/dL), hyperglycemia defined as fasting glucose ≥110 mg/dL or type 2 diabetes mellitus (DM2),^40^ hypertension (≥130/85 mm Hg or if under treatment). Mean arterial pressure (MBP) = ((2 × diastolic arterial pressure) + systolic arterial pressure)/3.

Glomerular filtration rate (GFR) was determined through a 24-hour creatinine clearance test and using the “modification of diet in renal disease” formula (MDRD) (GFR = 186 × (creatinine) − 1.154 × (age) − 0.203 or (× 0.742 in women)).^41^ Chronic renal failure (CRF) was assumed when the GFR was ≤60 mL/min/1.73 m^2^.

Demographic, clinical, and biochemical variables were reported with descriptive statistics: tendency and dispersion measures for continuous variables and proportions for dichotomous or nominal variables. The Mann–Whitney *U* test, Fisher test, ANOVA with Bonferroni correction, and the *χ*^2^ tests were used to compare continuous and categorical variables in each group; we considered statistical significance level of 0.05 (both sides). The Cochran Q, Pearson and Spearman correlation tests as well as linear regression models were carried out: dependent variables: Lep o AdipoQ serum values adjusted by independent variables (forward process): metabolic variables (BMI, MBP, glucose, total cholesterol, triglycerides, HDL-cholesterol values), and variables related to inflammatory join disease activity (number of swollen or painful joints, HAQ, and pain VAS scores). All statistical tests were performed using IBM SPSS Statistics 21.

## RESULTS

Forty-one patients with gout, mean age of 48.0 ± 12.9 years were included. Forty of them were males; 32 (78.1%) had tophaceous gout and in 80.5% of the patients we demonstrated monosodium urate crystals upon polarized light microscopy of the synovial fluid or tophi.

At baseline, the mean duration of disease was 12.6 ± 10.5 years; however, none of them received adequate treatment and therefore had evidence of disease inflammatory activity (frequent acute flares in the 6 months before baseline visit and swollen and tender joints as well as an increase in acute phase reactants) and also chronicity data (hyperuricemia, tophi and limited to motion joints). Almost one third of the patients had MetS or its comprising entities, the most common of which was hypertriglyceridemia, followed by hypertension and obesity; a third of the patients had CRF (Table 1).

**Table 1:**
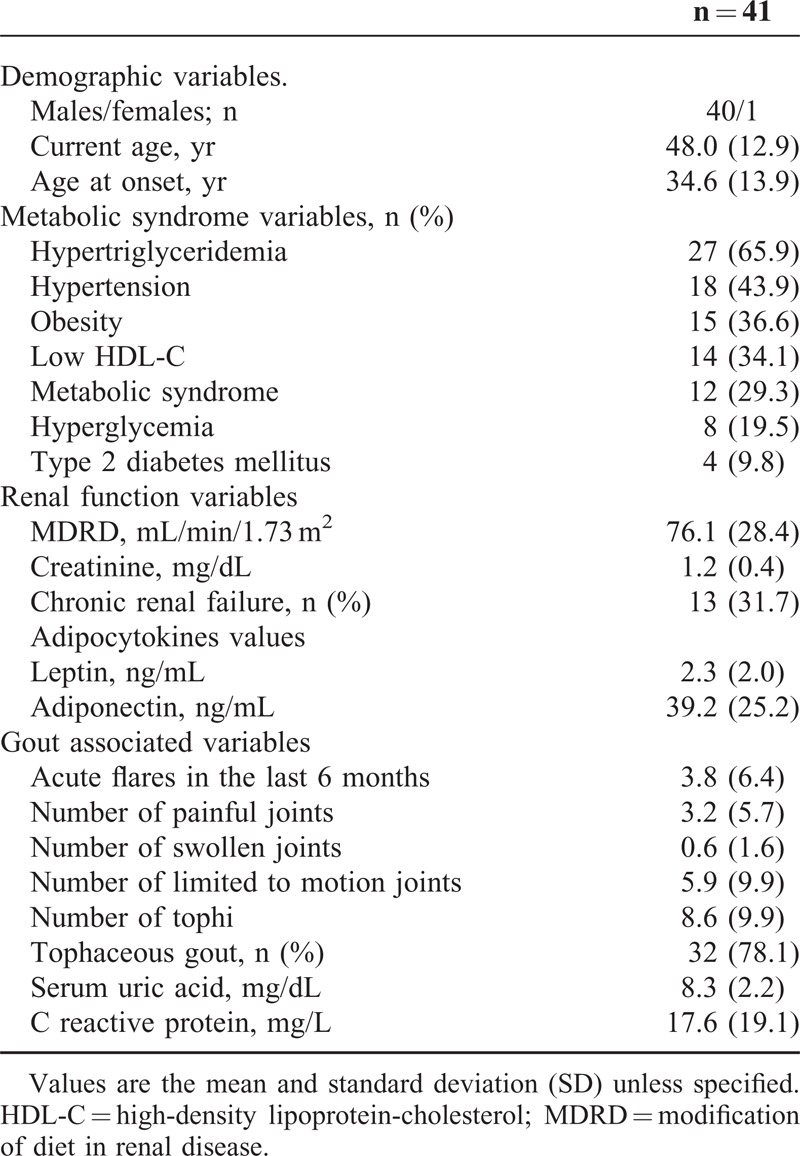
Baseline Demographic and Clinical Data

### Baseline Evaluation: Gout With MetS Versus Gout Without MetS

In the baseline evaluation, patients with gout and MetS had significantly greater levels of CRP and greater but not significantly levels of Lep. On the other hand, these same patients had a better renal function according to the 3 measures we employed for this variable (MDRD, serum creatinine and percentage of patients with CRF), but only serum creatinine levels had a tendency to be significantly different (Table 2).

**Table 2:**
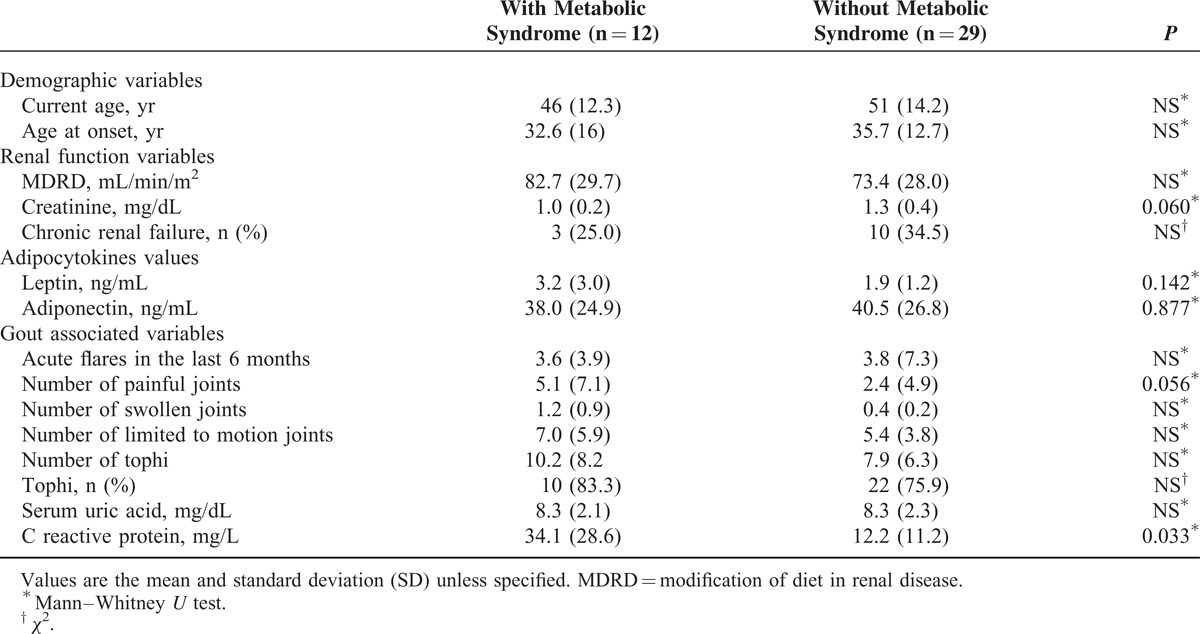
Comparison of Baseline Demographic and Clinical Data of Gout Patients With or Without Metabolic Syndrome

As expected, when comparing patients with MetS to those without it, the former had a greater frequency of hypertension, obesity, hyperglycemia, and dyslipidemia; however, we did not find significant differences in the related demographic or clinical variables related to gout or in the AdipoQ levels (Table 2).

### 6 and 12 Months Follow-Up

#### Gout

**FIGURE 1:**
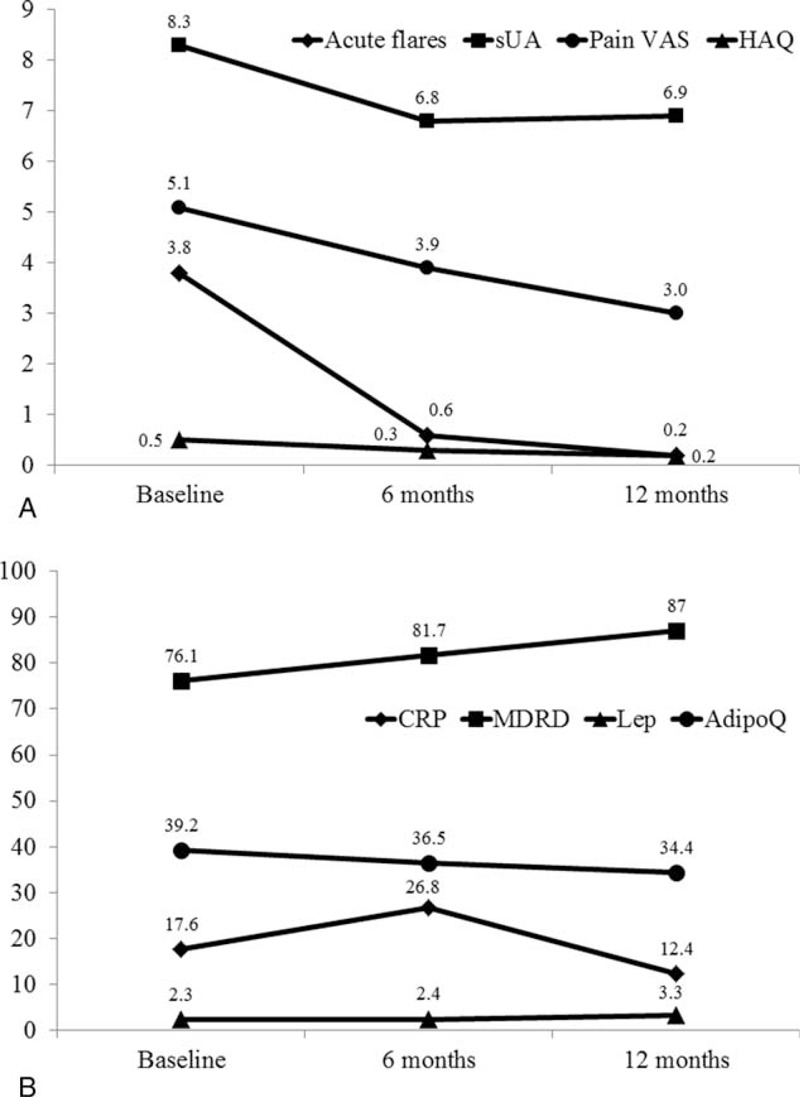
A, Improvement in gout clinical data (mean values). Baseline, 6 and 12-month evaluations. B, Gout para-clinical data and adipocytokine levels (mean values). Baseline, 6 and 12-month evaluations. Acute flares = number of flares in the last 6 months; AdipoQ = adiponectin (ng/mL); CRP = C reactive protein (mg/dL); HAQ = health assessment questionnaire score; Lep = leptin (ng/mL); MDRD = modified diet in renal disease (mL/min/1.73 m^2^); sUA = serum uric acid (mg/dL); VAS = visual analog scale.

We found significant improvement in the sUA values, MDRD, and in the variables evaluating joint pain and swelling at 6 and 12 months (Figure 1A and B). We observed that patients with MetS had higher baseline levels of CRP and at 6 months; these values steadily decreased, although this change was not significant (Table 3).

**Table 3:**
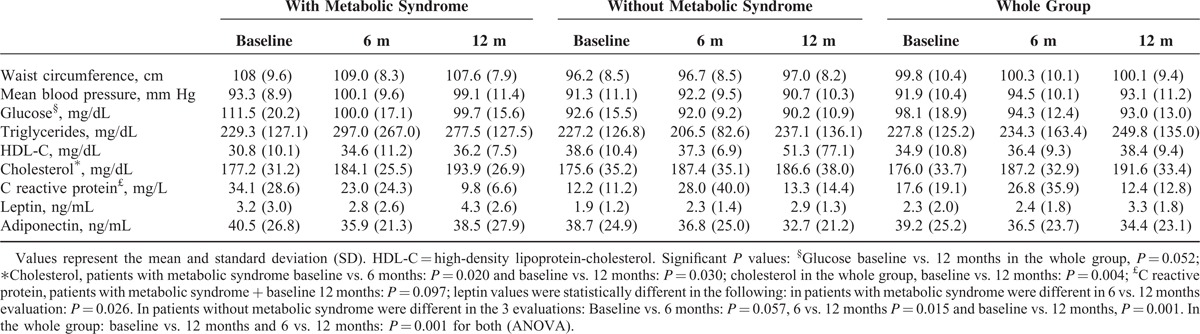
Comparison of Metabolic Variables. Baseline, 6 and 12 Months in Patients With or Without Metabolic Syndrome and in the Whole Group

After 1 year of follow-up, only 10 patients (24.4%) had sUA levels ≤6.0 mg/dL; treatment was individualized but in general terms consisted of starting regular treatment with allopurinol at a low dose followed by a steady increase: (300 ± 189.8 mg/day at the baseline visit to 450 ± 206 mg/day at 12 months). Forty patients received allopurinol and only 1 patient received probenecid (due to allergy to allopurinol); all patients received prophylaxis with colchicine (1 mg/day).

#### MetS

During the follow-up, we did not find significant improvement in the associated metabolic entities, our patients showed increase in total cholesterol and tryglicerides, maintained their waist circumference and MBP, and had slight improvement in HDL-C and glycaemia (Table 3). Although 57.9% of them (MetS patients) received bezafibrate, 5.1% fenofibrate, 10.5% received statins, 73.7% losartan or enalapril and metformin was prescribed to 26.3%. Enalapril or losartan were employed as antihypertensive treatment in those who required it, only 1 patient received diuretics and another was taking low-dose aspirin.

#### Clinical Status and Serum Adipocytokine Levels

The levels of adipocytokines, especially Lep, tended to increase both in the group of patients with MetS as well as overall, in the same way that metabolic abnormalities tended to persist or even worsen during follow-up (Table 4).

**Table 4:**
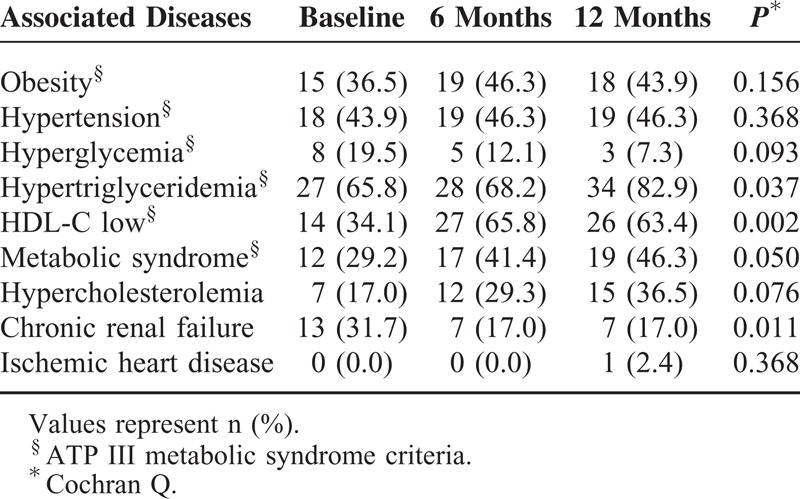
Percentage of Patients With Gout With Associated Diseases

After performing a linear correlation of the Leptin and AdipoQ values, there was no important correlation (*r* > 0.5) between other metabolic variables and those measuring the activity of gout.

During follow-up, a 54-year-old patient died due to a myocardial infarction; this patient had a history of intense tobacco and alcohol consumption, tophaceous gout for 30 years with an inadequate control, MetS (obesity, hypertension, and dyslipidemia), his baseline Lep and AdipoQ serum levels were similar to the values from other patients.

## DISCUSSION

This study evaluated the possible relationship between improvement in clinical gout and/or metabolic data and the serum concentration of adipocytokines in patients in whom regular treatment was initiated.

During follow-up with regular treatment, the patients improved significantly in all the variables directly related to gout as joint inflammatory activity and in the sUA levels; although only one fourth of them achieved sUA ≤6.0 mg/dL. Similar to other studies, we did not find relation among the concentration of adipocytokines and sUA levels.^32,33^

Lep values had relationship with obesity, MetS, and CRP, but not with gout-related variables and course during 6 and 12 months follow-up. These Lep and AdipoQ (especially Lep) values related to MetS course had been previously reported in patients with MetS, without gout.^42–45^

Interestingly CRP, a marker of systemic inflammatory state and considered a marker for joint “activity” as well as marker for metabolic and cardiovascular risk, tended to lower levels during follow-up in patients with gout associated with MetS, but not in patients without it.

Previous reports evaluating whether adipocytokines are protective or harmful in joint inflammatory diseases had been controversial. However, at the same time, some authors have reported that the presence of high levels of these adipocytokines must always be considered abnormal and may be associated with metabolic or inflammatory abnormalities; in addition, the diversity of cells containing receptors for these adipocytokines makes them scarcely specific and finally, their expression and functions seem to be different in different body tissues.^46^

Unfortunately, we were not capable of modifying most metabolic variables during 1 year of follow-up. The data are unsatisfactory because, excepting a tendency to lower glucose levels, the rest of the variables did not improve and there was even a significant increase in the total cholesterol levels; in addition, during follow-up we observed increase in the number of patients with low HDL-C values and MetS.

There are difficulties in compliance and adherence in patients with chronic diseases and especially in gout patients, in 1 study >80% of gout patients had partial and poor adherence to ULD therapy, the adherence to lifestyle modifications in them seems to be lesser, although not known.^47^

Probably, the sample size of this study, although adequate to evaluate clinical improvement, was not enough to evaluate minor changes in AdipoQ values. Finally, all the above results suggest that in patients with gout, the concentrations of Lep and AdipoQ are mainly related to the metabolic state and not the articular clinical activity itself.
